# THC and CBD Induce Heme Oxygenase-1-Dependent Cell Death and Trigger Mitochondrial Dysfunction in Human Melanoma and Cutaneous Squamous Cell Carcinoma Cells

**DOI:** 10.3390/antiox15030286

**Published:** 2026-02-26

**Authors:** Elisabeth Thamm, Felix Wittig, Bianca Hamann, Franziska Wendt, Steffen Emmert, Marcus Frank, Burkhard Hinz

**Affiliations:** 1 Institute of Pharmacology and Toxicology, Rostock University Medical Center, 18057 Rostock, Germany; 2 Clinic and Policlinic for Dermatology, Rostock University Medical Center, 18057 Rostock, Germany; 3 Electron Microscopy Center, Rostock University Medical Center, 18057 Rostock, Germany; 4 Department Life, Light and Matter, University of Rostock, 18059 Rostock, Germany

**Keywords:** cannabinoids, skin cancer, melanoma cells, cutaneous squamous cell carcinoma cells, cell death, heme oxygenase-1, mitochondria, oxygen consumption rate, respiratory chain complex proteins

## Abstract

In the search for new therapeutic strategies for the treatment of skin cancer, cannabinoids have become the focus of scientific interest. The present study investigated the effects of the phytocannabinoids Δ^9^-tetrahydrocannabinol (THC) and cannabidiol (CBD) on the viability, apoptosis, and mitochondrial function of human melanoma (A375) and cutaneous squamous cell carcinoma (SCC) cells (A431). Both cannabinoids caused a time- and concentration-dependent loss of viability and an upregulation of caspase-3/7 activity, associated with the induction of initiator caspases-8 and -9, PARP cleavage, and an increase in the autophagy marker LC3A/B-II. Inspired by the latest work on the dual role of heme oxygenase-1 (HO-1) in cell fate, the expression of this enzyme was examined and found to be upregulated at the mRNA and protein level by THC and CBD. Inhibition of HO-1 activity by tin protoporphyrin IX (SnPPIX) reduced the loss of viability caused by both cannabinoids, suggesting a cytotoxic rather than cytoprotective mediator role for this enzyme here. At the mitochondrial level, THC and CBD caused a reduction in membrane potential, a release of cytochrome c into the cytosol, and electron microscopically detectable mitochondrial damages. A more detailed functional analysis revealed an inhibition of mitochondrial oxygen consumption rate, accompanied by a decrease in various subunits of mitochondrial oxidative phosphorylation complexes. In conclusion, our data demonstrate a strong cytotoxic effect of THC and CBD on melanoma and cutaneous SCC cells involving mitochondrial apoptosis and mitochondrial dysfunction.

## 1. Introduction

Skin cancers are categorized into melanoma, which is the most aggressive form, and non-melanoma skin cancers, which include basal cell carcinoma (BCC) and squamous cell carcinoma (SCC), the most commonly diagnosed forms [1]. In recent years, major advances have been achieved in the drug treatment of skin cancer. In cases of inoperable melanoma, for example, immune checkpoint inhibitors (ICI), which block CTLA-4 (cytotoxic T-lymphocyte-associated protein 4), PD-1 (programmed cell death protein 1), PD-1 ligand 1 (PD-L1) and LAG-3 (lymphocyte activation gene 3), have been approved (for reviews, see [2,3]). Moreover, in the case of a mutation in the v-raf murine sarcoma viral oncogene homolog B (BRAF) kinase at position 600 (BRAF^V600^), targeted therapy with BRAF inhibitors and inhibitors of downstream to BRAF operating mitogen-activated extracellular signal-regulated kinase (MEK) can be employed (for reviews, see [4,5]). In cutaneous SCC, inhibitors of the PD-1/PD-L1 immune checkpoint axis are effective, as are inhibitors of the epidermal growth factor receptor (EGFR) [6,7]. However, these relatively new drug classes also have disadvantages, such as the dose-dependent inflammatory side effects of ICI [8,9] or the secondary resistance and a relatively short response duration of BRAF/MEK inhibitor therapies [10,11]. In this context, there is still an unmet need to improve the efficacy of skin cancer treatment and to overcome the drug resistance of existing pharmacotherapies.

Cannabinoids have emerged as a promising option for the treatment of skin cancer in preclinical studies, exerting anticancer effects at various stages of skin cancer development by inhibiting tumor growth, proliferation, invasion, and angiogenesis, as well as by inducing apoptosis and autophagy (for reviews, see [12,13]). With regard to the clinical effect of cannabinoids, the data on the systemic anticancer effect of nabiximols, a Cannabis extract consisting of equal amounts of the phytocannabinoids Δ^9^-tetrahydrocannabinol (THC) and cannabidiol (CBD), in high-grade glioblastomas [14] also give reason to hope that these compounds could be used as an add-on to existing chemotherapy for other tumor entities, including skin cancer.

However, the molecular mechanisms of the cannabinoid-induced death of various skin cancer cells have not yet been conclusively clarified in the preclinical studies conducted to date. This seems all the more important for drawing conclusions about possible synergistic or antagonistic effects with existing chemotherapies. For example, the enzyme heme oxygenase-1 (HO-1) appears to be a promising target, as recent studies have demonstrated that its upregulation plays a mediating role in the induction of apoptotic [15,16,17,18,19,20] and ferroptotic cell death of tumors [21,22,23,24,25,26,27,28,29,30], in contrast to its classic cytoprotective function. In the case of HO-1 induced by the non-psychoactive phytocannabinoid CBD in human endothelial cells, we have already shown that the enzyme triggers protective autophagy, but leads to apoptosis at higher CBD concentrations and thus also increased HO-1 levels [31]. Further investigations by our group and others have reported that HO-1 is also upregulated in cancer cells by phytocannabinoids [32] or endocannabinoids [33]. However, the role of HO-1 in cancer remains complex, with both beneficial and detrimental effects (for review, see [34]). In addition, studies are needed to investigate the influence of cannabinoids on the cellular energy metabolism of skin cancer cells as part of their antitumor effect and thus as a target for appropriate pharmacotherapy. Indeed, CBD is known to have a variety of effects on mitochondrial function (for review, see [35]).

In recent years, we have used various preclinical oncological assays to identify and characterize drug candidates that reduce the survival rate of both melanoma and cutaneous SCC cells [36,37,38]. In one of these studies, HO-1 was shown to mediate the tumor cell toxic effect in either cell type [37]. The present study was therefore conducted to investigate the effect of THC and CBD on the viability of melanoma (A375) and cutaneous SCC cells (A431), with particular consideration of the role of HO-1 and mitochondrial dysfunction in cannabinoid-induced cell death. The effects shown here demonstrate an involvement of HO-1 in this process and provide convincing evidence for profound mitochondrial damage, which partially becomes evident even at subtoxic concentrations of both cannabinoids.

## 2. Materials and Methods

### 2.1. Materials

(−)-trans-Δ^9^-THC (THC, dronabinol, #THC-135) was purchased from Lipomed (Wesel, Germany) and cannabidiol (CBD, #BN0124) was bought from Biotrend Chemikalien (Cologne, Germany). AM251 (#71670), AM630 (#10006974), R-1 methanandamide (R(+)-methanandamide, mAEA, #90070) and ruthenium red (RR, #14339) were obtained from Cayman Chemical (Ann Arbor, MI, USA). JWH-133 (#1343) was bought from Tocris, Bio-Techne (Wiesbaden-Nordenstadt, Germany) and capsazepine (#C191) was obtained from Sigma-Aldrich (Taufkirchen, Germany). Tin protoporphyrin IX dichloride (SnPPIX; #ALX-430-051-M005) was purchased from Enzo Life Sciences (Lörrach, Germany). Aprotinin, bromophenol blue, hydrogen peroxide solution (H_2_O_2_, 30%), luminol, orthovanadate, *p*-coumaric acid, phenylmethanesulfonyl fluoride (PMSF) and penicillin–streptomycin were obtained from Sigma-Aldrich (Taufkirchen, Germany). Leupeptin was bought from Biomol (Hamburg, Germany). Acetic acid, dimethyl sulfoxide (DMSO), ethylenediaminetetraacetic acid (EDTA), glycerol, glycine, hydrochloric acid 37% (HCl), sodium chloride (NaCl), sodium hydroxide (NaOH), Tris ultrapure and Tris hydrochloride (Tris HCl) were obtained from AppliChem (Darmstadt, Germany). Methanol was ordered from J. T. Baker (Griesheim, Germany) and aqua ad iniectabilia from Braun Melsungen (Melsungen, Germany). 4-(2-hydroxyethyl)-1-piperazineethanesulfonic acid (HEPES) and β-mercaptoethanol were bought from Ferak Berlin (Berlin, Germany). Acrylamide (Rotiphorese^®^ Gel, 30%), ammonium peroxydisulphate (APS), crystal violet, N,N,N′,N′-tetramethylethylenediamine (TEMED), sodium dodecyl sulfate (SDS) ultra pure, Tris ultrapure, Triton^®^ X-100 and Tween^®^ 20 were purchased from Carl Roth (Karlsruhe, Germany). Non-fat milk (NFM) powder was bought from Bio-Rad Laboratories (Munich, Germany). Dulbecco’s phosphate-buffered saline (DPBS) was obtained from PAN-Biotech (Aidenbach, Germany). High-glucose Gibco™ DMEM (4.5 g/L glucose, GlutaMAX™-I supplement and pyruvate; #31966021) and Gibco^TM^ Trypsin-EDTA were purchased from Thermo Fisher Scientific (Schwerte, Germany) and fetal bovine serum (FBS) superior from Bio&Sell (Feucht by Nürnberg, Germany).

### 2.2. Cell Culture

A375 (human melanoma cell line; #300110; RRID:CVCL_0132) and A431 cells (human skin epidermoid carcinoma cell line; #300112; RRID:CVCL_0037) were obtained from CLS Cell Lines Service (Eppelheim, Germany). Cells were frozen in large stocks in early passages and used within three months following thawing. Cells were cultured in DMEM (high glucose, GlutaMAX^TM^-I supplement, pyruvate) supplemented with 10% (*v*/*v*) heat-inactivated FBS Superior, 100 U/mL penicillin, and 100 μg/mL streptomycin (hereafter referred to as serum-containing DMEM) in a humidified incubator at 37 °C and 5% CO_2_. Incubation with test substances or their vehicles was performed in serum-free DMEM supplemented with 100 U/mL penicillin and 100 μg/mL streptomycin (hereafter referred to as serum-free DMEM) and was carried out after washing the cells with DPBS. The test substances were dissolved in aqua ad iniectabilia (RR), ethanol (THC, CBD, mAEA, JWH-133), DMSO (AM251, AM630, capsazepine), or 1 M sodium hydroxide (SnPPIX). The final concentration of solvents in the incubation media of cells treated with test substances and vehicle did not exceed 0.1% (*v*/*v*) for ethanol (except for experiments with mAEA, where 0.3% (*v*/*v*) was used) and/or 0.2% (*v*/*v*) DMSO and 1 mM for sodium hydroxide. The incubation media of cells treated with vehicle and/or test substance contained the same amount of solvent.

### 2.3. Metabolic Activity and Cell Survival Assay

A375 or A431 cells were seeded in 96-well plates at a density of 5000 cells per well in serum-containing DMEM, cultured for 24 h and then treated with the test substances in serum-free DMEM for the specified times.

Cell viability was analyzed by measuring the metabolic activity of the cells with the water-soluble tetrazolium salt WST-1 (4-[3-(4-iodophenyl)-2-(4-nitrophenyl)-2H-5-tetrazolio]-1,3-benzene disulfonate, Roche Diagnostics, Mannheim, Germany). To this end, WST-1 reagent was added in a final dilution of 1:10, which, after appropriate incubation, was cleaved to a soluble formazan dye. This reduction depends largely on the production of NAD(P)H in viable cells. The absorbance was then measured at 450 nm (wavelength correction at 690 nm) using a microplate reader (Infinite F200 Pro Tecan, Tecan Group, Männedorf, Switzerland).

In addition to the WST-1 test, crystal violet staining was also performed in special cases. The latter provides a direct correlation with the adherent viable cells and thus enables a direct assessment of cell survival and death [39]. For this purpose, the cells were fixed with ice-cold absolute ethanol overnight and then incubated in 100 µL crystal violet staining solution (0.1% (*w*/*v*) crystal violet in 10% (*v*/*v*) ethanol) for 30 min. The excess dye was then carefully rinsed off, the remaining dye was dissolved with 10% (*v*/*v*) acetic acid, and the absorbance at 570 nm was determined using a microplate reader (Infinite F200 Pro Tecan).

### 2.4. Caspase Activity Assays

A375 or A431 cells were seeded in 96-well plates at a density of 5000 cells per well, cultured for 24 h in serum-containing DMEM, and then incubated in serum-free DMEM with the appropriate test substance for the times indicated. Promega luciferase assays (Madison, WI, USA) were used to measure caspase activity (Caspase-Glo^®^ 3/7 assay, #G8091; Caspase-Glo^®^ 8 assay, #G8201; Caspase-Glo^®^ 9 assay, #G8211). For this purpose, the corresponding Caspase-Glo^®^ reagents were added to the wells in accordance with the manufacturer’s instructions and incubated in the dark at room temperature for 1 h. The luminescence was detected using a microplate reader (Infinite F200 Pro Tecan).

### 2.5. Determination of Mitochondrial Membrane Potential

The JC-10 Mitochondrial Membrane Potential Assay Kit (AAT Bioquest, Pleasanton, CA, USA) was employed to assess changes in mitochondrial membrane potential. Therefore, A375 or A431 cells were seeded in 96-well plates at a density of 15,000 cells per well and cultured for 24 h in serum-containing DMEM before treatment with THC, CBD, or vehicle in serum-free DMEM. After 24 h of incubation, the cells were washed with 100 µL DPBS. Then, 100 µL of a freshly prepared and pre-warmed JC-10 working solution (10 µM final concentration) in serum-free DMEM was added to the cells. The plate was incubated at 37 °C for 45 min and then washed with 100 µL DPBS. Finally, 100 µL DPBS was added to each well for measurement. Fluorescence was detected using a microplate reader with excitation/emission wavelengths of 485/535 nm (green fluorescence) and 535/595 nm (orange fluorescence). The data were blank-corrected (using wells containing cells without JC-10) and analyzed by calculating the ratio of fluorescence intensities at emission wavelengths of 595 nm (orange) and 535 nm (green).

### 2.6. Seahorse XFe Analysis

Oxygen consumption rate (OCR) was measured with the Seahorse XFe24 Analyzer (Agilent Technologies, Waldbronn, Germany) according to the manufacturer’s instructions. For this purpose, A375 and A431 cells were seeded on Seahorse XF24 plates (Agilent Technologies) at a density of 25,000 cells per well and incubated in serum-containing DMEM for 24 h. Following DPBS washing, cells were treated with THC, CBD, or vehicle in serum-free DMEM for 24 h. At the beginning of the Seahorse XFe analysis, the medium was changed to Seahorse XF medium, pH 7.4 (#103575-100, Agilent Technologies), supplemented with 10 mM glucose, 2 mM glutamine and 1 mM pyruvate. The Seahorse XF medium remained on the cells for another 1 h. In accordance with the manufacturer’s instructions and our own cell line-specific preliminary tests, compounds from the Seahorse XF Cell Mito Stress Test Kit (#103015-100, Agilent Technologies) were then added to the wells (port A: 1.5 μM oligomycin (ATP synthase inhibitor); port B: 2.5 μM (for A375 cells) or 1 µM (for A431 cells) FCCP (carbonyl cyanide 4-(trifluoromethoxy)phenylhydrazone; uncoupling agent that collapses the proton gradient and disrupts the mitochondrial membrane potential); port C: 0.5 μM rotenone (mitochondrial complex I inhibitor) and 0.5 μM antimycin A (mitochondrial complex III inhibitor)). OCR per well was normalized to the respective amount of protein, which was determined after the experiment. Therefore, 10 µL of lysis buffer (50 mM HEPES, 150 mM NaCl, 1 mM EDTA, 1% [*v*/*v*] Triton^®^ X-100, 10% [*v*/*v*] glycerol, 10 µg/mL aprotinin, 1 µg/mL leupeptin, 1 mM orthovanadate and 1 mM PMSF) was added to each well and the lysates were collected. Protein concentration was determined using the Pierce™ BCA Protein Assay Kit (Thermo Fisher Scientific).

Basal respiration, ATP-linked respiration, spare respiratory capacity, and proton leak were calculated using the Seahorse XF Cell Mito Stress Test Report Generator (Agilent Technologies). Non-mitochondrial respiration, defined as the minimal respiration remaining after rotenone/antimycin A injection, was automatically subtracted from the basal respiration, spare respiratory capacity, and proton leak values.

### 2.7. Total Cellular Protein Isolation

A375 or A431 cells were seeded in 6-well plates at a density of 200,000 cells per well and cultured for 24 h in serum-containing DMEM before treatment with compounds in serum-free DMEM. At the end of each incubation, the supernatants were collected, the cells washed with DPBS and detached with trypsin-EDTA. In order to obtain a higher total protein quantity, the corresponding cells of a treatment group from an independent experiment were combined from 3 wells each. To this end, supernatants, DPBS washing solutions and detached cells from each treatment group were combined and pelleted by centrifugation (200× *g*, 4 °C) for 5 min. Next, the cell pellet was washed with DPBS and centrifuged again for 5 min (200× *g*, 4 °C). Thereafter, the cell pellet was mixed with lysis buffer (50 mM HEPES, 150 mM NaCl, 1 mM EDTA, 1% [*v*/*v*] Triton^®^ X-100, 10% [*v*/*v*] glycerol, 10 µg/mL aprotinin, 1 µg/mL leupeptin, 1 mM orthovanadate, 1 mM PMSF), incubated overnight at −20 °C, and then centrifuged for 5 min (20,817× *g*, 4 °C). The resulting supernatant, which contained the total cellular protein, was collected and stored for further protein analysis. Protein concentrations were determined using the Pierce™ BCA Protein Assay Kit.

### 2.8. Mitochondrial Protein Isolation

A375 or A431 cells were seeded, treated and harvested as in the whole-cell protein isolation experiments. In order to achieve a higher total protein quantity, the detached cells of a treatment group from an independent experiment were combined from 12 wells each. The cell pellet obtained after centrifugation (7 min, 500× *g*, 4 °C) was washed in 0.9% NaCl and then centrifuged again (5 min, 200× *g*, 4 °C). The Qproteome Mitochondria Isolation Kit (Qiagen, Hilden, Germany) was used to isolate mitochondria and obtain the cytosolic protein fraction according to the manufacturer’s instructions for standard preparation. To extract the mitochondrial protein, the resulting mitochondrial fraction was treated with lysis buffer, centrifuged, and collected in a similar manner to the protocol for isolating total cellular protein.

### 2.9. Western Blot Analysis

Equal amounts of protein were separated on an 8% (PARP analysis), 12% (GPX4 analysis) or 15% (all other proteins) SDS–polyacrylamide gel, transferred to a nitrocellulose membrane and incubated for 1 h in 5% (*w*/*v*) NFM in Tris-buffered saline containing 0.1% (*v*/*v*) Tween^®^ 20 (TBS-T buffer). Following washing with TBS-T buffer, membranes were incubated overnight at 4 °C with primary antibodies in 1% (*w*/*v*) NFM (applies for cytochrome c, OxPhos antibodies) or 5% (*w*/*v*) NFM (applies for GPX4, HO-1, HO-2, LC3A/B, PARP, and cleaved PARP antibodies) in TBS-T buffer.

The OxPhos Human WB Antibody Cocktail (#45-8199, RRID: AB_2533836), detecting representative subunits of mitochondrial oxidative phosphorylation (OXPHOS) complexes I–V at their expected apparent molecular weights (NDUFB8 ~18 kDa, SDHB ~29 kDa, UQCRC2 ~48 kDa, COX2 ~22 kDa, ATP5A ~54 kDa), was obtained from Thermo Fisher Scientific. Antibodies against LC3A/B (~16 kDa [LC3-I], ~14 kDa [LC3-II]; #4108, RRID: AB_2137703), PARP (~116 kDa; #9532, RRID: AB_659884), cleaved PARP (~89 kDa; #32563, RRID: AB_2799024), and cytochrome c (~14 kDa; #11940, RRID: AB_2637071) were purchased from Cell Signaling Technology (Frankfurt/Main, Germany). Antibodies against GPX4 (predicted ~22 kDa, observed <20 kDa; #ab125066, RRID: AB_10973901) and VDAC1/Porin + VDAC3 (~31 kDa; #ab14734, RRID: AB_443084) were obtained from Abcam (Berlin, Germany). HO-1 (~32 kDa; #ADI-SPA-894, RRID: AB_10631417) and HO-2 (~36 kDa; #ADI-SPA-897, RRID: AB_10615082) antibodies were from Enzo Life Sciences, and GAPDH (~36 kDa; #G9545, RRID: AB_796208) and β-Actin (~42 kDa; #A5441, RRID: AB_476744) from Sigma-Aldrich. After washing with TBS-T buffer, the membranes were incubated with secondary antibodies coupled to horseradish peroxidase (anti-rabbit antibody, #7074; RRID: AB_2099233, or anti-mouse antibody, #7076, RRID: AB_330924; both from Cell Signaling Technology) in 1% (*w*/*v*) NFM in TBS-T buffer for 1 h at room temperature. To visualize antibody binding, a chemiluminescence solution (100 mM Tris hydrochloride pH 8.5, 1.25 mM luminol, 200 µM *p*-coumaric acid, 0.09% [*v*/*v*] H_2_O_2_) was added and signal detection was performed using the ChemiDoc XRS gel documentation system (Bio-Rad Laboratories, Munich, Germany). Signal intensity was quantified using Quantity One 1-D Analysis Software Version 4.6.6 (Bio-Rad Laboratories). The signal of a specific protein band was normalized to the signal of the loading control. As loading controls, the protein contents of VDAC (for proteins in mitochondrial fractions), GAPDH (for proteins in cytosolic fractions) or β-actin (for total cell protein) were related to the respective target protein analyzed. It is noteworthy that the antibody VDAC1/Porin + VDAC3 (#ab14734, RRID: AB_443084), which was used to normalize mitochondrial proteins, recognizes both VDAC1 and VDAC3, which have a similar calculated molecular weight of 31 kDa and can only be distinguished by their post-translational modifications in gel electrophoresis [40]. In the present study, normalization was performed consistently using the upper band with the higher expression level, which is likely to be VDAC1, the most abundant isoform in most tissues [41].

Finally, the protein contents determined this way were calculated relative to the vehicle control. The Precision Plus Protein™ Dual Color Standard (Bio-Rad Laboratories) was used to determine the molecular weight of the bands. Upon completion of the analysis, the membranes were stripped and reprobed.

### 2.10. Quantitative Reverse Transcriptase Polymerase Chain Reaction (qRT-PCR)

A375 or A431 cells were seeded and cultured as in the experiments for isolating total cell proteins. Thereafter, the cells were incubated with THC, CBD, or vehicle in serum-free DMEM for 6 h. At the end of each cell incubation, the cells were harvested according to the protein isolation experiments with trypsin-EDTA. In order to obtain a higher total RNA quantity, the detached cells of a treatment group from an independent experiment were combined from 2 wells each. Cells were pelleted by centrifugation (500× *g*, 4 °C) for 5 min. The cell pellet was then washed with DPBS and centrifuged again for 5 min (500× *g*, 4 °C). Following total RNA isolation using the RNeasy Mini Kit (Qiagen), mRNA levels of HO-1, HO-2, and peptidylprolyl-isomerase A (PPIA, housekeeping gene) were determined by real-time RT-PCR on an Applied Biosystems 7500 Fast Real-Time PCR System (Applied Biosystems, Carlsbad, CA, USA) using the Applied Biosystems^®^ TaqMan^®^ RNA-to-CT™ 1-Step Kit (Thermo Fisher Scientific). Primers and probes for human PPIA (Assay ID: Hs999904_m1; VIC-MGB), HO-1 (Assay ID: Hs01110251_m1; FAM-MGB) and HO-2 (Assay ID: Hs00157969_m1; FAM-MGB) were Applied Biosystems^®^ TaqMan^®^ Gene Expression Assay products (Thermo Fisher Scientific). HO-1 and HO-2 mRNA were normalized to PPIA mRNA.

### 2.11. Electron Microscopy

A375 or A431 cells were seeded at a density of 200,000 cells per well in 6-well-plates and cultured in serum-containing DMEM for 24 h. Thereafter, the cells were incubated with THC, CBD, or vehicle in serum-free DMEM for 24 h and then treated as described for the protein extraction experiments. For each treatment group, detached cells from 6 wells were combined. The cell pellets were fixed in a solution of 2% glutaraldehyde and 1% paraformaldehyde in 0.1 M phosphate buffer (pH 7.3) and stored at 4 °C until further processing for embedding. After washing twice in 0.1 M phosphate buffer, the cells were mixed with prewarmed 2% low melting agarose (Sigma-Aldrich) in 0.05 M HEPES buffer at 40 °C, collected by centrifugation, and, after hardening, enclosed as pellets in the agarose. The specimen blocks were then post-fixed for 2 h in a 1% osmium tetroxide solution (Carl Roth) and, after washing in distilled water, dehydrated in an ascending series of acetone. Resin infiltration started with overnight incubation in a 1:1 mixture of acetone and Epon resin (48% Epon 812, 30% methyl nadic anhydride, 20.7% 2-dodecenylsuccinic acid anhydride, 1.3% 2,4,6-tris(dimethylaminomethyl)phenol; all components from Serva, Heidelberg, Germany), and then for 4 h in pure Epon resin. Specimen blocks were transferred to silicone rubber moulds with fresh resin and cured in an oven at 60 °C for 2 days. Further processing included trimming of the resin blocks (Leica EM Trim2, Leica Microsystems, Wetzlar, Germany) and subsequent sectioning on an ultramicrotome (Ultracut S, Reichert, Wien, Austria) with a diamond knife (Diatome, Nidau, Switzerland). For visualization and selection of areas for ultrastructural examination, semithin sections with a thickness of 0.5 µm were stained with toluidine blue. Ultrathin sections with a thickness of approximately 50–70 nm were transferred to copper grids and stained with uranyl acetate and lead citrate before being examined with a Zeiss EM902 transmission electron microscope operated at 80 kV (Carl Zeiss, Oberkochen, Germany). Digital images were acquired with a side-mounted 1x2k FT-CCD Camera (Proscan, Scheuring, Germany) using iTEM camera control and imaging software (iTEM version number 1187, Olympus Soft Imaging Solutions, Münster, Germany). Additional images were obtained using a FEI Talos L120C G2 transmission electron microscope operated at 120 kV equipped with a 4x4k CMOS Ceta camera and Velox imaging software version 3.2.1 (FEI Deutschland GmbH, part of Thermo Fisher Scientific, Dreieich, Germany).

### 2.12. Statistics

All values are given as mean ± standard error of the mean (SEM). Calculations were performed using GraphPad Prism 9.3.0 or an updated version (GraphPad Software, San Diego, CA, USA). Comparisons between multiple groups and a vehicle group were performed using one-way analysis of variance (ANOVA) with Dunnett’s post hoc test. When groups were compared to more than one group of interest, a one-way ANOVA with a Bonferroni post-hoc test was performed. In the latter case, for reasons of clarity, the determination of statistical significance was limited to the groups of interest. *p* values of ≤0.05 were considered significant.

## 3. Results

### 3.1. THC and CBD Decrease Viability of A375 and A431 Cells in a Concentration- and Time-Dependent Manner

In a first set of experiments, the concentration- and time-dependent effects of THC and CBD on the viability of A375 melanoma and A431 cutaneous SCC cells were investigated by determining the metabolic activity using the WST-1 assay. For comparison, the hydrolysis-stable anandamide (AEA) analog R(+)-arachidonyl-1′-hydroxy-2′-propylamide (R-1 methanandamide, R(+)-methanandamide, mAEA) [42], a potent CB_1_ receptor agonist [43,44] and TRPV1 agonist [45], and the selective CB_2_ receptor agonist JWH-133 [46] were included in these experiments.

**Figure 1:**
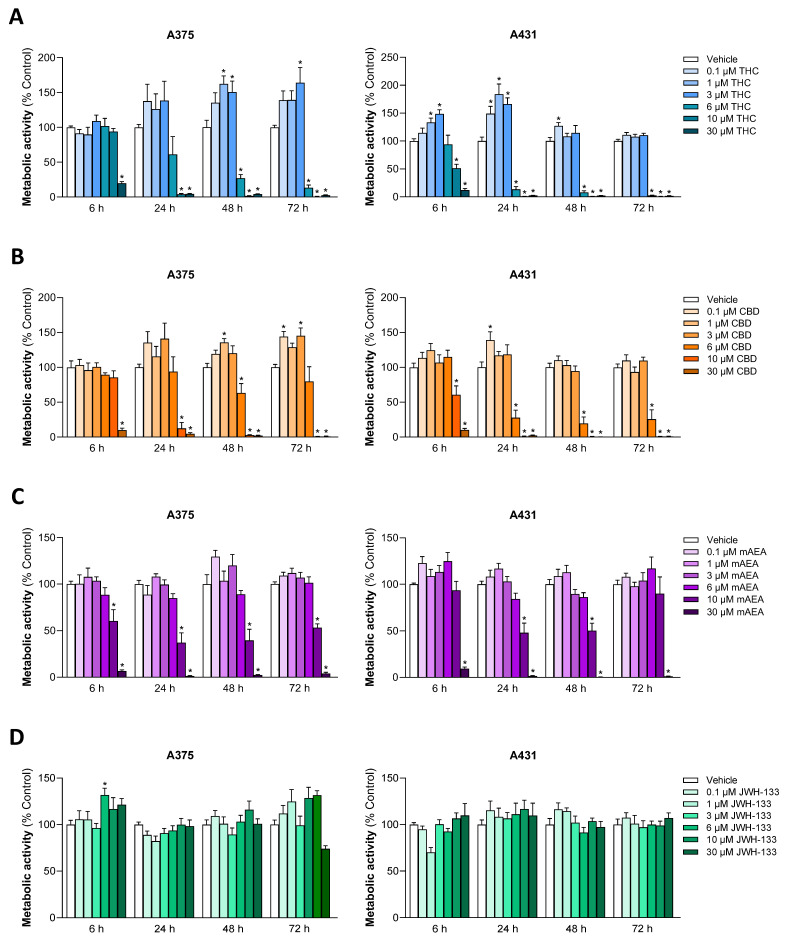
Effect of THC (**A**), CBD (**B**), mAEA (**C**), and JWH-133 (**D**) on metabolic activity of A375 and A431 cells. Cells were incubated with the respective cannabinoid at the indicated concentrations for the indicated times. The values given are based on WST-1 assays. All percentage values shown refer to the respective time-matched vehicle control, which was set to 100%. The data are mean values ± SEM of *n* = 8–9 per group from 3 independent experiments. * *p* ≤ 0.05 vs. corresponding vehicle control; one-way ANOVA with Dunnett’s post hoc test.

For THC (Figure 1A) and CBD (Figure 1B), a significant decrease in viability was already evident after 6 h of treatment at 30 µM (A375) or 10 and 30 µM (A431), which was also achieved after 24 h when 10 µM (A375) and 6 µM (A431) of the respective cannabinoid was used. Finally, after 48 h, significant viability losses could be detected in both cell lines in the presence of 6 µM of either cannabinoid. In view of the slightly higher sensitivity of A431 cells, incubation times of 48 h for A375 cells and 24 h for A431 cells were used in subsequent WST-1 and caspase-3/7 analyses to characterize the effects of THC and CBD.

In the case of mAEA (Figure 1C), significant losses of viability were recorded starting at concentrations of 10 µM after 6 h (A375) or 24 h (A431) of incubation. A unique case was JWH-133 (Figure 1D), which at no time caused a significant reduction in the metabolic activity of A375 and A431 cells.

Remarkably, stimulation of metabolic activity was also observed in the lower cannabinoid concentration range (Figure 1), although this could not be linked to increased proliferation. Accordingly, when testing a corresponding non-toxic concentration range (0.1 to 3 µM), no changes in cell count corresponding to the results in the crystal violet assay could be detected (Supplementary Figure S1), suggesting a rather cell stress-induced increase in metabolic activity. On the other hand, the crystal violet assay showed a significant decrease in the cell counts of A375 and A431 after incubation with concentrations of 6 µM or higher for THC and CBD. In the case of mAEA, significant reductions were observed starting at 10 µM in A375 cells and at 6 µM in A431 cells. Again, JWH-133 had no effect, even at a test concentration of 30 µM (Supplementary Figure S1).

### 3.2. THC and CBD Cause a Concentration- and Time-Dependent Activation of the Executioner Caspases-3 and -7 in A375 and A431 Cells

**Figure 2:**
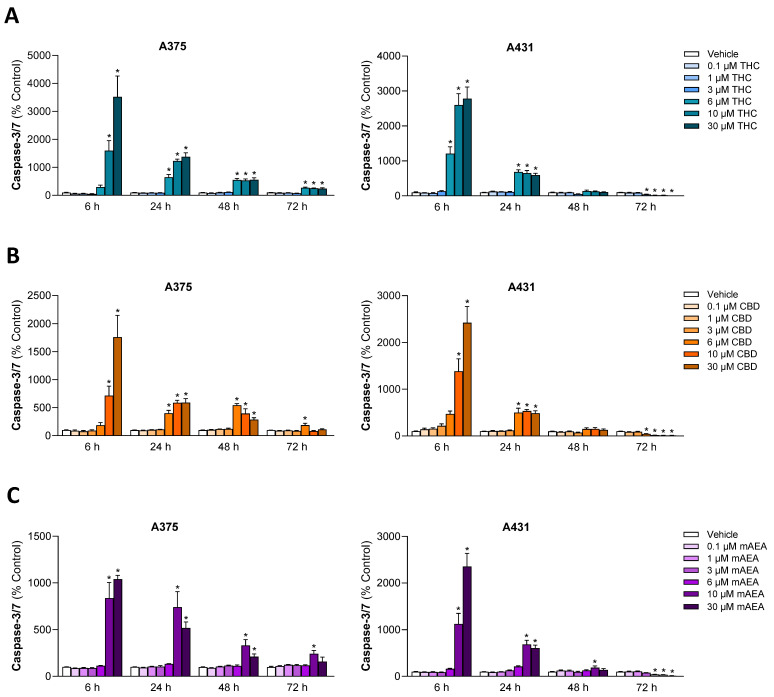
Effect of THC (**A**), CBD (**B**), and mAEA (**C**) on activation of caspases-3/7 in A375 and A431 cells. Cells were incubated with the respective cannabinoid at the indicated concentrations for the indicated times. The values given are based on caspase-3/7 activity assays. All percentage values shown refer to the respective time-matched vehicle control, which was set to 100%. The data are mean values ± SEM of *n* = 9 per group from 3 independent experiments. * *p* ≤ 0.05 vs. corresponding vehicle control; one-way ANOVA with Dunnett’s post hoc test.

In order to obtain initial evidence of apoptotic cell death, the effect of THC and CBD, here still with the inclusion of mAEA for comparative purposes, on the activity of the executioner caspases-3 and -7 was investigated (Figure 2). For all cannabinoids examined, an early induction of the effector caspases was observed after 6 h. At this early time point, significant upregulation was observed for both THC and CBD at 10 µM or higher in either cell line, and in the case of THC in A431 cells, already at 6 µM. At concentrations of 6 to 30 µM THC and CBD, significant induction was still detectable after 48 h (A375) and 24 h (A431). In the case of mAEA, significant upregulations were limited to concentrations of 10 and 30 µM.

### 3.3. Investigation of the Receptor Dependency of the Effects of THC and CBD on Metabolic Activity and Caspase-3/7 Activity

To demonstrate a possible involvement of cannabinoid receptors in the effects caused by THC and CBD, further experiments were carried out with receptor antagonists, each used at a concentration of 1 µM, which has been reported to block the cannabinoid effects at CB_1_, CB_2_ or TRPV1 in various studies [47,48,49,50,51,52,53,54,55,56]. Examination of the CB_1_ receptor antagonist AM251 and the CB_2_ receptor antagonist AM630, alone or in combination, showed no significant inhibitory effect on the decrease in metabolic activity and increase in caspase-3/7 activity triggered by THC, CBD and the reference substance mAEA (Supplementary Figure S2). Measurable, albeit insignificant, inhibitions of the viability-reducing effect of THC were observed in A431 cells when AM251 was administered and in A431 and A375 cells when AM251 and AM630 were given in combination, but without comparable effects at the caspase-3/7 activity level. Viability and apoptosis parameters of A375 and A431 cells treated with CBD, a known TRPV1 agonist [57], were also not altered by the TRPV1 antagonist capsazepine (Supplementary Figure S3). Finally, none of the receptor antagonists tested had a significant impact per se on metabolic activity (Supplementary Figures S2A,G and S3A,C) and caspase-3/7 activation (Supplementary Figures S2D,J and S3B,D).

To further assess the potential contribution of TRP channels to the viability-reducing effects of THC and CBD, cells were treated with ruthenium red, a non-selective inhibitor of several Ca^2+^-permeable TRP channels, including TRPV1–TRPV4 and TRPA1. Notably, CBD has also been identified as a potent agonist of TRPA1 and TRPV2 [58]. However, at 10 µM, a concentration commonly used to inhibit CBD-induced TRP channel activation [59,60], ruthenium red neither attenuated the CBD- nor THC-induced loss of cell viability (Supplementary Figure S4).

### 3.4. Effect of THC and CBD on Further Apoptosis Markers as Well as on Markers of Autophagy and Ferroptosis in A375 and A431 Cells

**Figure 3:**
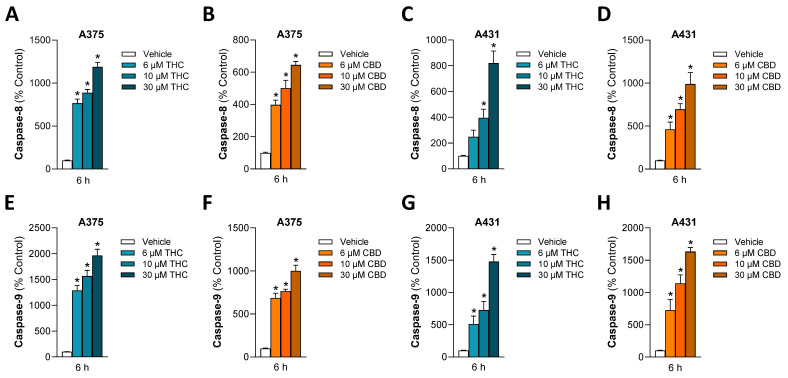
Effect of THC and CBD on activation of caspase-8 (**A**–**D**) and caspase-9 (**E**–**H**) in A375 and A431 cells. Cells were incubated with THC or CBD at the indicated concentrations for 6 h. The values given are based on caspase activity assays. All percentage values shown refer to the respective time-matched vehicle control, which was set to 100%. The data are mean values ± SEM of *n* = 9 per group from 3 independent experiments. * *p* ≤ 0.05 vs. corresponding vehicle control; one-way ANOVA with Dunnett’s post hoc test.

With the focus on THC and CBD, subsequent experiments were conducted with these phytocannabinoids only. To confirm the apoptosis induction shown before, further parameters of programmed cell death were recorded. In this context, the initial focus was on caspases-8 and -9 as initiator caspases of the extrinsic and intrinsic apoptosis pathways, respectively, which converge at the end of the caspase cascade in the activation of effector caspases-3 and -7 [61]. After a 6-h incubation of A375 and A431 cells with THC or CBD, an activity analysis showed a significant upregulation of the initiator caspases-8 and -9 in both cell lines (Figure 3).

**Figure 4:**
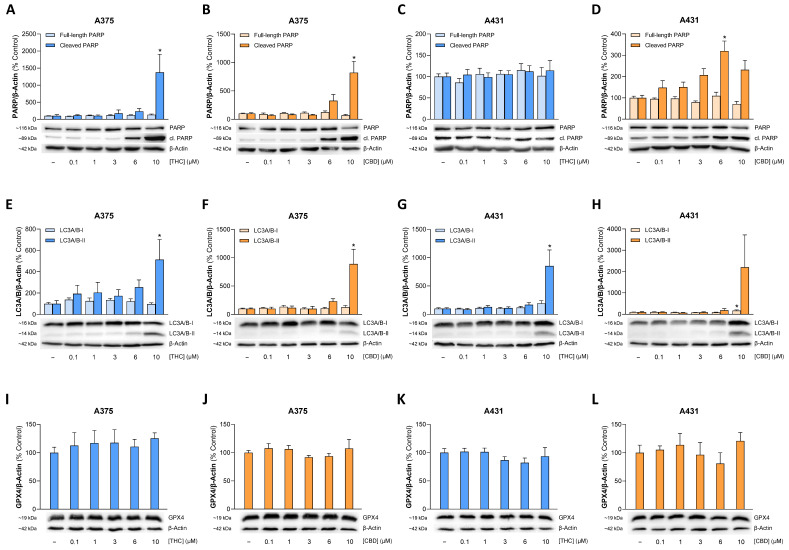
Effect of THC and CBD on PARP cleavage (**A**–**D**), LC3-I to LC3-II conversion (**E**–**H**), and GPX4 expression (**I**–**L**) in A375 and A431 cells. The cells were incubated with THC or CBD at the indicated concentrations for 24 h. The values given in the bar charts are based on densitometric analyses of the blots. PARP, cleaved PARP (cl. PARP), LC3A/B-I, LC3A/B-II and GPX4 levels were normalized to β-actin. All percentage values shown refer to the respective vehicle control, which was set to 100%. The blots shown are representative. The data are mean values ± SEM of *n* = 3 (**I**–**L**), *n* = 4 (**D**,**F**,**G**), *n* = 5 (**A**,**B**,**E**,**H**) or *n* = 6 (**C**) independent experiments. * *p* ≤ 0.05 vs. corresponding vehicle control; one-way ANOVA with Dunnett’s post hoc test.

Moreover, cleavage of poly(ADP-ribose) polymerase (PARP), a downstream target of effector caspases, was investigated in THC- or CBD-treated skin cancer cells. Thereby, an increase in the cleaved 89 kDa PARP fragment could be detected in most cases in A375 and A431 cells (Figure 4A,B,D). An exception was observed in THC-treated A431 cells, which on average showed only a 1.14-fold increase in cleaved PARP after 24-h incubation with 10 µM THC compared to vehicle-treated cells (Figure 4C).

The two phytocannabinoids also triggered a concentration-dependent upregulation of the autophagy marker light chain 3 microtubule-associated protein A/B (LC3A/B)-II in both cell lines (Figure 4E–H), while no degradation of the ferroptosis repressor glutathione peroxidase 4 (GPX4) was detectable (Figure 4I–L).

### 3.5. THC and CBD Induce Upregulation of HO-1 but Not HO-2 Expression in A375 and A431 Cells

**Figure 5:**
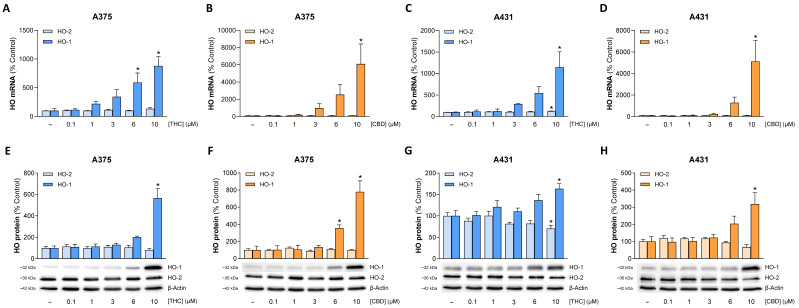
Effect of THC and CBD on the expression of HO-1 and HO-2 at the mRNA (**A**–**D**) and protein (**E**–**H**) level in A375 and A431 cells. Cells were incubated with THC or CBD at the indicated concentrations for 6 h (**A**–**D**) or 24 h (**E**–**H**). The values given in the bar charts are based on quantitative RT-PCR (**A**–**D**) or densitometric analyses of the blots (**E**–**H**). HO mRNA levels were normalized to PPIA mRNA levels and HO protein levels to β-actin. All percentage values shown refer to the respective vehicle control, which was set to 100%. The blots shown are representative. The mRNA and protein data are mean values ± SEM of *n* = 3 (**A**–**D**), *n* = 7 (**E**), *n* = 5 (**F**) or *n* = 6 (**G**,**H**) independent experiments. * *p* ≤ 0.05 vs. corresponding vehicle control; one-way ANOVA with Dunnett’s post hoc test.

On the basis of findings that showed a role for HO-1 upregulation in the induction of apoptosis [15,16,17,18,19,20,31], the expression modulation of this enzyme by THC and CBD was examined next. This showed a concentration-dependent induction of expression at the mRNA and protein level in both skin cancer cell lines examined (Figure 5). On the other hand, no significant modulation of the HO-2 isoenzyme was found in the corresponding samples.

### 3.6. Inhibition of HO-1 Activity with SnPPIX Reverses THC- and CBD-Induced Loss of Viability and Interferes with THC-Induced Caspase-3/7 Activation in A375 and A431 Cells

**Figure 6:**
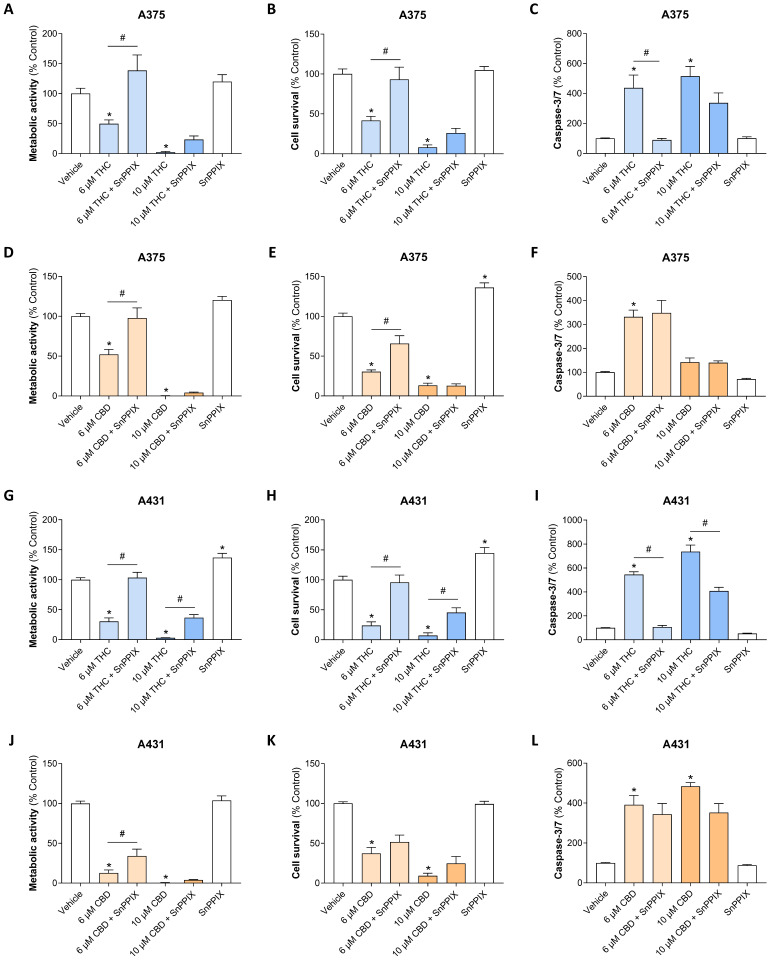
Influence of the HO-1 inhibitor SnPPIX on the decrease in metabolic activity (**A**,**D**,**G**,**J**) and cell number (**B**,**E**,**H**,**K**) as well as on the increase in caspase-3/7 activity (**C**,**F**,**I**,**L**) mediated by THC or CBD in A375 and A431 cells. Cells were pre-treated with SnPPIX (25 µM) or its vehicle for 1 h, followed by a 48-h (A375 cells) or 24-h (A431 cells) co-incubation with the indicated concentrations of THC or CBD or its vehicle. The data are mean values ± SEM of *n* = 9 from 3 independent experiments (**A**–**J**) or *n* = 12 from 4 independent experiments (**K**,**L**). * *p* ≤ 0.05 vs. corresponding vehicle control; # *p* ≤ 0.05 vs. corresponding THC- or CBD-treated group; one-way ANOVA with Bonferroni’s post hoc test.

To demonstrate the functional relevance of the HO-1 upregulation by THC and CBD, inhibitor experiments were carried out with the HO-1 inhibitor SnPPIX (Figure 6). Protoporphyrin-based inhibitors of HO-1 such as SnPPIX have been successfully used in a number of studies to confirm the role of HO-1 upregulation in induced cell death [15,16,21,22,23,24,25,26,27,29,31,37].

Corresponding studies were carried out to determine metabolic activity using the WST-1 assay, cell survival by crystal violet staining, and caspase-3/7 activity. Particularly for THC, a convincing inhibition of all these parameters was observed in the presence of the HO-1 inhibitor, which was complete when a submaximally toxic THC concentration of 6 µM was used. Likewise, SnPPIX was able to inhibit the CBD-induced effects. These changes were mainly observed in A375 cells at the level of metabolic activity and cell survival and, as with THC, when using a submaximally toxic concentration of 6 µM. On the other hand, no inhibition of CBD-induced caspase-3/7 activity could be determined in A375 cells. It should be noted that in some cases, SnPPIX also led to a significant increase in cellular survival rate (Figure 6E,H) or metabolic activity (Figure 6G) in the absence of a cannabinoid.

### 3.7. THC and CBD Increase Mitochondrial HO-1 Levels, Decrease Mitochondrial Membrane Potential, Elevate Cytosolic Cytochrome c Levels, and Cause Structural Mitochondrial Changes in A375 and A431 Cells

Given that both HO-1 induction and increased caspase-9 activity suggest mitochondrial involvement in cannabinoid-induced apoptotic cell death, subsequent investigations focused on corresponding mitochondrial alterations by THC and CBD.

**Figure 7:**
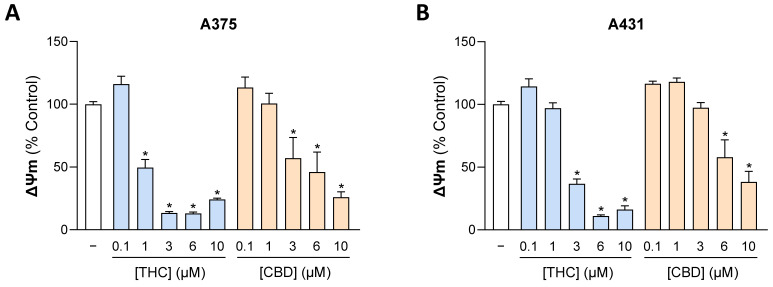
Effect of THC and CBD on mitochondrial membrane potential (ΔΨm) of A375 (**A**) and A431 cells (**B**). The cells were incubated with THC or CBD at the indicated concentrations for 24 h. ΔΨm was assessed using the JC-10 fluorescence ratio (orange to green) as described in Section 2 and expressed relative to the vehicle control (set to 100%). Data are mean values ± SEM of *n* = 9 from 3 independent experiments. All percentage values shown refer to the respective vehicle control, which was set to 100%. * *p* ≤ 0.05 vs. corresponding vehicle control; one-way ANOVA with Dunnett’s post hoc test.

To measure changes in mitochondrial membrane potential, JC-10, a lipophilic cationic dye that can selectively enter mitochondria, was used (Figure 7). Here, in most cases, a significant effect in the sense of a reduced membrane potential could be observed even at non-toxic concentrations of 1 µM (Figure 7A for THC) or 3 µM (Figure 7A for CBD, Figure 7B for THC) of the respective cannabinoid (compare with Figure 1A,B). JC-10 ratios (orange/green) at 10 µM THC were slightly higher than at 6 µM, likely reflecting reduced green fluorescence from cytotoxicity that partially masks mitochondrial depolarization. 

**Figure 8:**
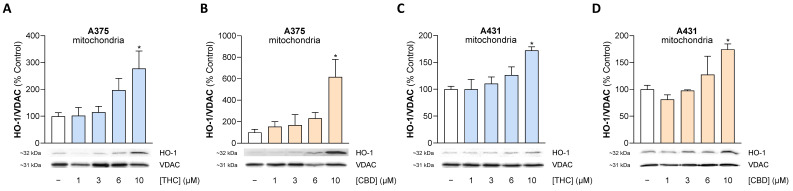
Effect of THC and CBD on mitochondrial HO-1 protein levels in A375 (**A**,**B**) and A431 cells (**C**,**D**). The cells were treated with the indicated concentrations of THC or CBD for 24 h. Thereafter, the corresponding proteins in the mitochondrial fractions were determined using Western blot analysis. The values given in the bar charts are based on densitometric analyses of the blots. Mitochondrial HO-1 was normalized to VDAC. All percentage values shown refer to the respective vehicle control, which was set to 100%. The blots shown are representative. In (**B**) the same VDAC blot is shown as in Figure 12D, and in (**C**) as in Figure 9C, as the same membranes were stripped and reprobed for different target proteins. The data are mean values ± SEM of *n* = 4 (**A**–**C**) or *n* = 3 (**D**) independent experiments. * *p* ≤ 0.05 vs. corresponding vehicle control; one-way ANOVA with Dunnett’s post hoc test.

The translocation of HO-1 to mitochondria has been described in response to severe cellular stress conditions including oxidative stress [62]. Therefore, the protein concentrations of HO-1, which were increased in the total cell lysates upon incubation with THC and CBD (Figure 5E–H), were also determined in the mitochondrial protein fractions. Here, after treatment with THC and CBD, measurable (6 µM concentrations) or significant (10 µM concentrations) inductions were observed in both cell lines (Figure 8). Overall, a comparatively higher upregulation was observed in the mitochondrial fractions of the A375 cells.

**Figure 9:**
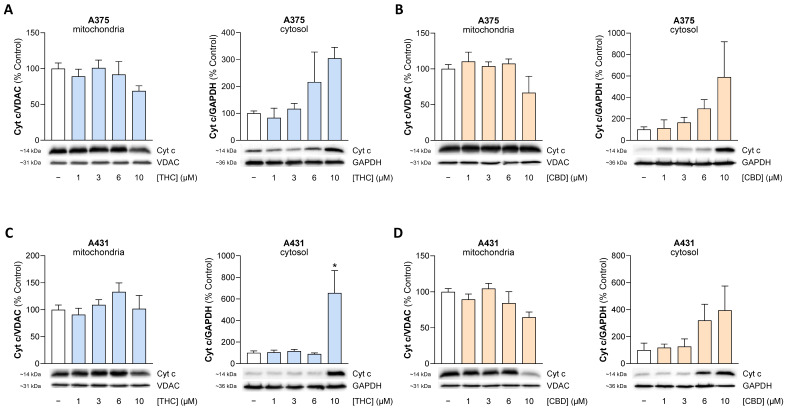
Effect of THC and CBD on the release of mitochondrial cytochrome c (Cyt c) into the cytosol of A375 (**A**,**B**) and A431 cells (**C**,**D**). The cells were incubated with THC or CBD at the indicated concentrations for 24 h. The values given in the bar charts are based on densitometric analyses of the blots. Cyt c levels were normalized on VDAC in mitochondrial fractions and on GAPDH in cytosolic fractions. The blots shown are representative. In (**A**) the same VDAC blot is shown as in Figure 12B, in (**C**) as in Figure 8C, and in (**D**) as in Figure 12H, as the same membranes were stripped and reprobed for different target proteines. The data are mean values ± SEM of *n* = 4 ((**A**,**B**,**D**) left graph each; (**C**)) or *n* = 3 ((**A**,**B**,**D**) right graph each) independent experiments. All percentage values shown refer to the respective vehicle control, which was set to 100%. * *p* ≤ 0.05 vs. corresponding vehicle control with Dunnett’s post hoc test.

Finally, a release of mitochondrial cytochrome c into the cytosol of A375 and A431 cells was caused by both THC and CBD (Figure 9), indicating mitochondrial dysfunction and explaining the previously observed activation of initiator caspase-9 and effector caspases-3 and -7.

**Figure 10:**
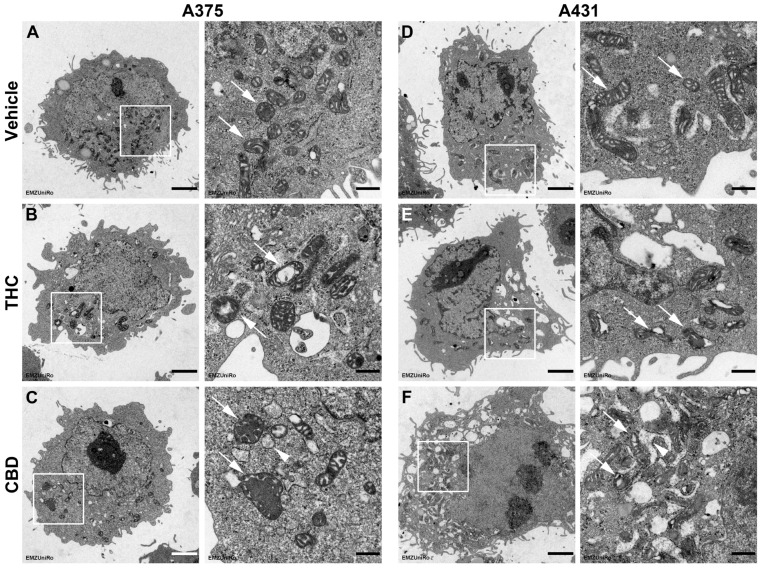
Influence of THC and CBD on the mitochondrial structure of A375 and A431 cells. Representative images from the transmission electron microscopy of A375 (**A**–**C**) and A431 cells (**D**–**F**) treated for 24 h with vehicle (**A**,**D**), 6 µM THC (**B**,**E**) or 6 µM CBD (**C**,**F**). The images on the right of a treatment group (scale bar: 500 nm) are enlarged views of the areas outlined by white boxes in the corresponding left image (scale bar: 2 µm). The structures highlighted with white arrows in the enlarged views represent intact mitochondria (vehicle) vs. damaged mitochondria (THC, CBD), while white arrowheads mark altered mitochondria-associated ER membranes (CBD).

Consistent with the biochemical and biophysical changes induced by THC and CBD indicative of mitochondrial dysfunction, structural mitochondrial alterations were confirmed by electron microscopy (Figure 10). In A375 cells (Figure 10A–C) and in A431 cells (Figure 10D–F), pronounced mitochondrial damage was evident following THC and CBD treatment. The arrows in the images of the cannabinoid groups indicate structural changes in the mitochondria, including membrane disorganization, e.g., widened and disrupted cristae, leading to a loss of structural integrity. In addition, treatment with CBD led to slight swelling of the adjacent endoplasmic reticulum (ER) (arrowheads in Figure 10C,F), suggesting impairment of the mitochondria-associated ER membrane (MAM), which is involved in the regulation of oxidative stress (for review, see [63]). In A431 cells (Figure 10D–F), structural damage of mitochondria was comparatively less pronounced after treatment with THC and CBD but remained clearly detectable. Interestingly, damage appeared to be more severe in this cell line after CBD treatment than after THC exposure.

### 3.8. THC and CBD Decrease the Oxygen Consumption Rate of A375 and A431 Cells

**Figure 11:**
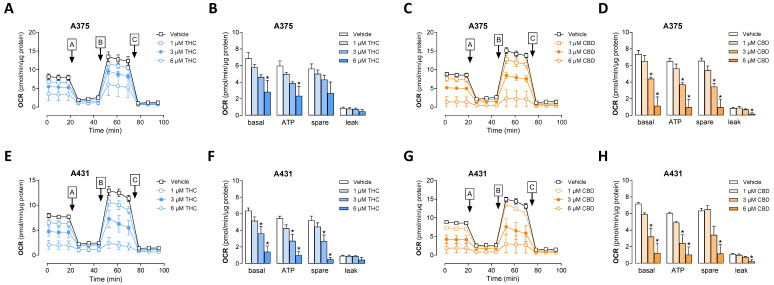
Effect of THC and CBD on oxygen consumption rate (OCR) of A375 (**A**–**D**) and A431 cells (**E**–**H**). The cells were incubated with THC or CBD at the indicated concentrations for 24 h. A mitochondrial stress test was then carried out and OCR values were determined using the Seahorse XFe24 Analyzer. Therefore, oligomycin (port A), FCCP (port B) and antimycin A/rotenone (port C) were loaded into the respective ports of the sensor cartridges and released into the wells at the specified times, as marked by enclosed uppercase letters with arrows in the time-course panels. From this assay, time courses of OCR in both cell lines treated with THC (**A**,**E**) or CBD (**C**,**G**) and calculations of basal respiration (basal), ATP-linked respiration (ATP), spare respiratory capacity and proton leak (**B**,**D**,**F**,**H**) are shown. The data represent mean values ± SEM of *n* = 3 per group from 3 independent experiments. * *p* ≤ 0.05 vs. corresponding vehicle control; one-way ANOVA with Dunnett’s post hoc test.

In the next series of experiments, we examined whether THC and CBD suppress mitochondrial energy production in the form of intracellular adenosine triphosphate (ATP), essential for tumor development. To this end, the oxygen consumption rate (OCR) was determined as an indicator of mitochondrial respiration using a mitochondrial stress test. In addition to basal respiration, this assay also quantifies ATP-linked respiration, maximal respiratory capacity, spare respiratory capacity, and non-mitochondrial respiration. The OCR curves shown in Figure 11A,C,E,G result from the time-delayed addition of artificial modulators of oxidative phosphorylation. Using this approach, it was shown that both THC and CBD cause a concentration-dependent reduction in basal respiration, ATP-linked respiration and spare respiratory capacity, indicating a strong impairment of cellular respiration (Figure 11B,D,F,H). Proton leak also showed reduced values at a THC and CBD concentration of 6 µM each.

### 3.9. THC and CBD Cause a Decrease in Specific Proteins of the Mitochondrial Oxidative Phosphorylation System in A375 and A431 Cells

**Figure 12:**
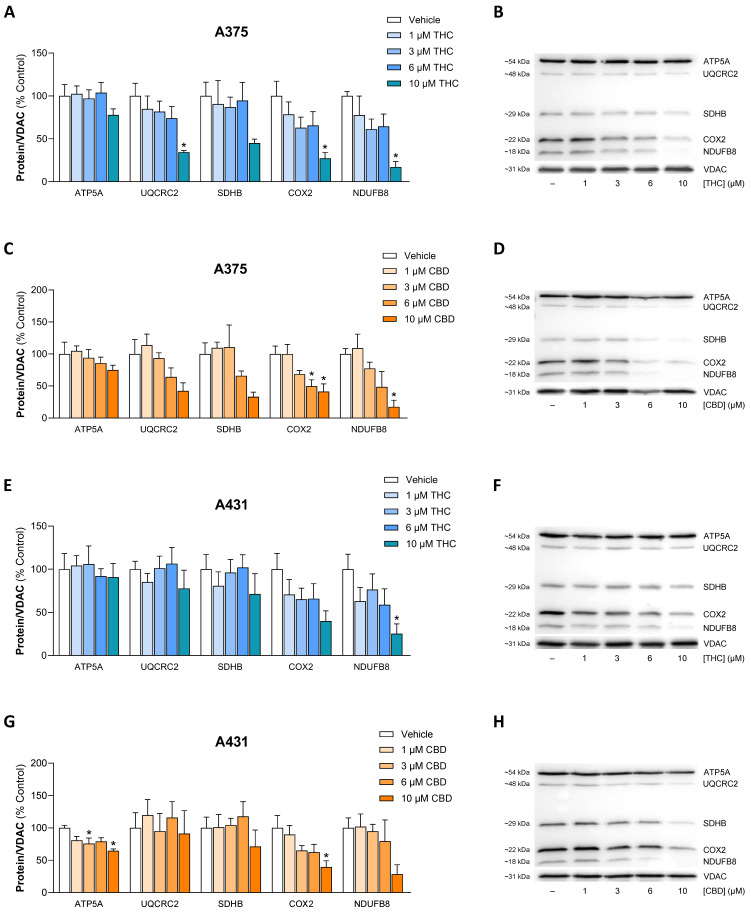
Effect of THC and CBD on the concentrations of subunits of mitochondrial respiratory chain complexes of A375 (**A**–**D**) and A431 cells (**E**–**H**). The cells were treated with the indicated concentrations of THC or CBD for 24 h. Thereafter, the corresponding proteins in the mitochondrial fractions were determined using Western blot analysis. The values given in the bar charts are based on densitometric analyses of the blots. Mitochondrial proteins were normalized to VDAC. All percentage values shown refer to the respective vehicle control, which was set to 100%. The blots shown are representative. In (**B**) the same VDAC blot is shown as in Figure 9A, in (**D**) as in Figure 8B, and in (**H**) as in Figure 9D, as the same membranes were stripped and reprobed for different target proteines. The data are mean values ± SEM of *n* = 4 independent experiments. * *p* ≤ 0.05 vs. corresponding vehicle control; one-way ANOVA with Dunnett’s post hoc test.

To further clarify the observed reduction in OCR, the influence of THC and CBD on the concentrations of specific subunits of the respiratory chain complexes in protein extracts from isolated mitochondria was investigated using an OxPhos antibody set. The following proteins were included in this analysis: NADH:ubiquinone oxidoreductase subunit B8 (NDUFB8, subunit of complex I), succinate dehydrogenase complex iron-sulfur subunit B (SDHB, subunit of complex II), ubiquinol-cytochrome c reductase core protein 2 (UQCRC2, subunit of complex III), cytochrome c oxidase subunit 2 (COX2, *MT-CO2*, subunit of complex IV) and mitochondrial ATP synthase F1 subunit alpha (ATP5A, subunit of complex V). The results are shown in Figure 12. In many cases, concentration-dependent reductions induced by THC or CBD were observed. At the highest concentration of 10 µM, THC and CBD triggered a reduction of more than 50% in the protein concentrations of NDUFB8, SDHB, UQCRC2 and COX2 in A375 cells, while in A431 cells the corresponding reductions were limited to NDUFB8 and COX2.

In order to prove or exclude the influence of cannabinoid-induced increased mitochondrial HO-1 on mitochondrial subunits of respiratory chain complexes, inhibitor experiments were also carried out here using the HO-1 inhibitor SnPPIX (Supplementary Figure S5). However, the inhibitory effects observed in these experiments, i.e., of CBD on NDUFB8 and COX2 in both cell lines and of THC on COX2 in both cell lines and NDUFB8 in A431 cells, were not reversed by SnPPIX.

## 4. Discussion

The present study demonstrates pronounced cytotoxic effects of the phytocannabinoids THC and CBD on melanoma and cutaneous SCC cells, indicating a prominent involvement of mitochondria-triggered apoptosis and mitochondrial dysfunction. There are several results that support this notion. Firstly, both cannabinoids caused a time- and concentration-dependent reduction in cell viability, which was associated with the activation of the two initiator caspases-8 and -9, as well as elevated activity of the effector caspases-3 and -7. Consistent with the activation of caspase-9, an increased release of mitochondrial cytochrome c into the cytosol and a reduction of the mitochondrial membrane potential were detected. Finally, evidence of mitochondrial dysfunction was provided by findings showing decreases in OCR and certain electron transport chain proteins after treatment of cells with THC or CBD.

The cytotoxic effect reported here for THC and CBD is not new and has been previously described in melanoma cells for THC [64,65,66] and CBD [66,67,68,69]. Likewise, a corresponding effect of CBD on cutaneous SCC is known [70,71], but not, to our knowledge, for THC. On the other hand, the selective CB_2_ receptor agonist JWH-133 appears to have a special position, as it did not cause any significant toxicity on A375 and A431 cells in our experiments. These findings are consistent with those of another study in which no effect of JWH-133, tested in concentrations up to 5 µM, on the viability of melanoma cells (COLO38, OCM-1) could be shown [72]. In the same study, WIN 55,212-2 proved to be a potent antimitogenic substance whose effect was not reversed by the CB receptor antagonists AM251 and AM630, suggesting a receptor-independent effect of this cannabinoid. Another explanation for the lack of cytotoxic effect for JWH-133 may lie in a reduced density of CB_2_ receptors in the cell lines examined. In fact, RT-PCR analysis showed that A375 cells express mRNA for the cannabinoid receptors CB_1_ and TRPV1, but not for CB_2_ [73]. On the other hand, JWH-133 has been found to exert antimigratory and anti-adhesive effects on A2058 melanoma cells [74] and to exhibit tumor-regressive and anti-angiogenic effects in vivo in B16 melanoma cell xenografts [64].

Interestingly, in several cases in the present study, subtoxic cannabinoid concentrations induced an increase in metabolic activity, although increased proliferation was ruled out by unchanged cell numbers in the crystal violet assay. It is noteworthy that other studies have also observed comparable biphasic (hormetic) metabolic responses for various compounds and nanoparticles in the WST-1 assay, with stimulation of metabolic activity at low concentrations and inhibition at higher concentrations [75,76,77,78,79,80,81]. A possible explanation may be that mild cellular stress increases electron flux through plasma membrane redox systems, enhancing the reduction of extracellular tetrazolium acceptors to formazan, independent of oxygen coupled mitochondrial respiration [82,83].

As long as cannabinoids have been preclinically investigated for anti-tumor effects, there have also been divergent findings that attribute a corresponding action to different cannabinoid receptors or to receptor-independent events (for reviews, see [84,85]). In the present investigation, a non-significant partial inhibition of THC-induced viability reduction was achieved by the CB_1_ antagonist AM251 or its combination with AM630, whereas this effect was not observed at the level of caspase-3/7 activity. A substantial involvement of cannabinoid receptors in the melanoma-toxic and SCC-toxic effect of the tested cannabinoids could thus be excluded. This is also supported by our reference experiments, which show that the tumor cell-toxic effects of mAEA, a potent CB_1_ receptor agonist [43,44], were not prevented by AM251. Additional inhibitor experiments using capsazepine, a TRPV1 antagonist, and ruthenium red, a broad-spectrum TRP channel inhibitor, excluded a contribution of TRP channels such as TRPV1, TRPV2, and TRPA1 to the cytotoxic effects of CBD, a potent activator of these ion channels.

The early induction of the initiator caspases-8 and -9 as classical mediators of the extrinsic and intrinsic apoptosis pathway, respectively, demonstrated for THC and CBD, is consistent with findings from cannabinoid studies conducted by other groups [72,86,87,88]. As a matter of fact, both initiator caspases can work together to activate the effector caspases-3 and -7, but also by caspase-8-mediated cleavage of the pro-apoptotic Bcl2 family member Bid, which is involved in the release of cytochrome c as part of the intrinsic signaling pathway [89,90]. In our hands, the typical features of a mitochondrial apoptosis pathway [91], consisting of a decrease in mitochondrial membrane potential and the release of cytochrome c, could be demonstrated for both cannabinoids. Concordant with the strong upregulation of caspase-3/7 activity, cleavage of the caspase substrate PARP was detected in most cases, except in A431 cells treated with THC. This may be due to the fact that the optimal time point was not achieved in this particular case. However, it could also indicate a general absence of this process. In fact, cases of early and late apoptosis have been described in the literature, measured using annexin V staining and associated with mitochondrial bifurcations and a reduction in mitochondrial membrane potential, but without PARP cleavage [92]. Finally, we were able to show that cannabinoids lead to an activation of autophagy in melanoma as well as cutaneous SCC cells. The latter has been attributed a cytotoxic role in the sense of non-canonical autophagy-mediated apoptosis by some authors, at least with regard to the toxic effect of THC on melanoma cells [65].

Regarding the mechanism of the skin-cancer-toxic effect of THC and CBD, this is the first study to demonstrate a functionally relevant increase in HO-1 mRNA and protein expression by cannabinoids in A375 and A431 cells. Accordingly, inhibition of HO-1 enzymatic activity by SnPPIX led to reversal of the THC-induced decrease in metabolic activity and cell survival, as well as THC-induced caspase-3/7 activity. In the case of CBD, corresponding inhibitory effects of SnPPIX on viability were registered, albeit not at the caspase level.

In line with the surprising finding of cytotoxic potential for HO-1, an enzyme originally thought to be cytoprotective only, a number of consistent findings have been reported in recent years confirming [15,16,17,18,19,20] or at least suggesting [93,94,95,96,97,98,99,100] a pro-apoptotic role for HO-1 in tumor cells. Even more frequently, HO-1 upregulation has been associated with ferroptosis induction by various compounds [21,22,23,24,25,26,27,28,29,30], although this type of cell death is rather unlikely for THC and CBD in our study due to the lack of effect on cellular GPX4 levels. Overall, HO-1 appears to play a complex role in tumors, manifesting either in cytoprotective effects associated with harmful consequences or in beneficial cytotoxic effects with regard to tumor control (for review, see [34]). Remarkably, HO-1 can also play a protumoral role at other levels of cancer progression (for reviews, see [101,102]), as indicated, for example, by its promoting effect on tumor angiogenesis in pancreatic cancer [103].

In addition to mitochondrial apoptosis being characterized by a decrease in the mitochondrial membrane potential and the release of cytochrome c into the cytosol, an increase in mitochondrial HO-1 by THC and CBD was also detected. It is known that HO-1 translocated to mitochondria maintains its enzymatic activity there (for review, see [104,105]). A corresponding translocation of HO-1 to the mitochondria has been described in the literature as a response to severe cellular stress conditions such as oxidative stress [62] or hypoxia [62]. Similarly, increased mitochondrial HO-1 activity was observed in mice treated with lipopolysaccharide [106]. Mitochondrial HO-1 has also been associated with elevated reactive oxygen species production and increased mitochondrial recruitment of the autophagy markers LC3 and Drp-1 [62].

To assess functional mitochondrial bioenergetics, a Seahorse XF Mito Stress Test was performed in A375 and A431 cells, which revealed a concentration-dependent decrease in various OCR parameters investigated by the cannabinoids studied. Here, decreases in OCR were recorded even at concentrations of both cannabinoids that were not toxic in the viability test, which is consistent with previous observations with both cannabinoids in glioblastoma cells [32]. In accordance with the functional data, THC and CBD led to a reduction in various subunits of electron transport chain complexes. This concerned, among others, complexes I and IV, for which reductions were also described in glioblastoma cells by the combination of both phytocannabinoids [32]. In contrast to the HO-1-dependent effect of THC and CBD on cell death, the reduction of specific proteins in the respiratory chain by the two cannabinoids was HO-1-independent. Accordingly, the HO-1 inhibitor SnPPIX could not reverse the respective cannabinoid-induced decreases in intramitochondrial protein concentrations. These investigations were included because previous studies have shown that HO-1 exerts both stimulatory [107,108,109] and inhibitory effects [62,106,110] on mitochondrial biogenesis.

This study has some limitations that need to be discussed. First, the protective effect of SnPPIX against cannabinoid-induced cell death was markedly more pronounced at submaximal, not yet fully cytotoxic concentrations of 6 μM THC and CBD, whereas it was substantially weaker (for THC) or largely absent (for CBD) when near-maximally cytotoxic 10 μM concentrations were tested. Similar findings were obtained in human and canine glioma cells [111] under otherwise different experimental conditions (including serum-containing medium), where the pan-caspase inhibitor Z-VAD-FMK protected cells at lower cytotoxic CBD concentrations (7.5–10 µg/mL, ≈24–32 µM) but failed to protect them at the maximum cytotoxic CBD concentration of 20 µg/mL (≈64 µM), suggesting that once a fully executed cytotoxic response is triggered, cells can no longer be rescued by inhibitory intervention. Second, protoporphyrin-based HO-1 inhibitors have been repeatedly described as successful tools for confirming the role of HO-1 upregulation in induced cell death [15,16,21,22,23,24,25,26,27,29,31,37]. As with all small molecules, however, off-target effects are also possible here [112], suggesting that future studies should also be conducted using genetic approaches. Third, it should be noted that CBD has been shown to target mitochondria via multiple mechanisms, including direct interaction with VDAC1, disruption of mitochondrial Ca^2+^ homeostasis and mitochondrial permeability transition pore (mPTP) opening [113,114,115,116]. These objectives were not addressed in detail again in the present study.

## 5. Conclusions

In summary, this study shows for the first time that inhibition of HO-1 may abrogate cannabinoid-induced tumor cell death. Moreover, the data presented here revealed a prominent role for mitochondria in cannabinoid-mediated melanoma and cutaneous SCC cell death. In addition to demonstrating the characteristics of mitochondrial cell death, significant and profound changes in mitochondrial bioenergetics could be proven for the cannabinoids examined. A further intensive preclinical study of cannabinoids as potential drugs for treating skin cancer is strongly recommended. This is particularly important as there are currently no randomized clinical trials investigating their efficacy and safety in melanoma or cutaneous SCC. Thus, it is also still too early to define the optimal formulation or route of administration.

## Data Availability

The original contributions presented in this study are included in the article/Supplementary Materials. Further inquiries can be directed to the corresponding author.

## Supplementary Materials

The following supporting information can be downloaded at: https://www.mdpi.com/article/10.3390/antiox15030286/s1, Figure S1: Concentration-dependent effects of THC (**A**,**E**), CBD (**B**,**F**), mAEA (**C**,**G**), and JWH-133 (**D**,**H**) on cell number of A375 and A431 cells. Figure S2: Influence of the CB_1_ antagonist AM251 and the CB_2_ antagonist AM630 on the decrease in metabolic activity as well as on the increase in caspase-3/7 activity mediated by THC (6 µM), CBD (6 µM), or mAEA (10 µM) in A375 (**A**–**F**) and A431 cells (**G**–**L**). Figure S3: Influence of the TRPV1 antagonist capsazepine (Capsa) on the decrease in metabolic activity and the increase in caspase-3/7 activity mediated by CBD (6 µM) in A375 (**A**,**B**) and A431 cells (**C**,**D**). Figure S4: Influence of the broad-spectrum TRP channel inhibitor ruthenium red (RR) on the decrease in cell number mediated by THC or CBD in A375 (**A**) and A431 cells (**B**). Figure S5: Investigation of the impact of SnPPIX on THC and CBD effects regarding the concentrations of subunits of mitochondrial respiratory chain complexes of A375 (**A**–**E**,**K**) and A431 cells (**F**–**J**,**L**).
