# Supplementary material for: THC and CBD Induce Heme Oxygenase-1-Dependent Cell Death and Trigger Mitochondrial Dysfunction in Human Melanoma and Cutaneous Squamous Cell Carcinoma Cells
Source: Antioxidants (Basel). 2026 Feb 26;15(3):286. doi: 10.3390/antiox15030286 (PMC13024539; doi:10.3390/antiox15030286)
Supplement: Supplementary file 1 [file antioxidants-15-00286-s001.zip › antioxidants-3970802-supplementary.pdf]

antioxidants

MDPI

Supplementary Materials

THC and CBD Induce Heme Oxygenase-1-Dependent Cell Death and Trigger Mitochondrial Dysfunction in Human Melanoma and Cutaneous Squamous Cell Carcinoma Cells

Elisabeth Thamm [1], Felix Wittig [1], Bianca Hamann [1], Franziska Wendt [1], Steffen Emmert [2], Marcus Frank [3,4] and Burkhard Hinz [1,*]

[1] Institute of Pharmacology and Toxicology, Rostock University Medical Center, 18057 Rostock, Germany
[2] Clinic and Policlinic for Dermatology, Rostock University Medical Center, 18057 Rostock, Germany
[3] Electron Microscopy Center, Rostock University Medical Center, 18057 Rostock, Germany
[4] Department Life, Light and Matter, University of Rostock, 18059 Rostock, Germany
* Correspondence: burkhard.hinz@med.uni-rostock.de

 https://doi.org/10.3390/antiox15030286

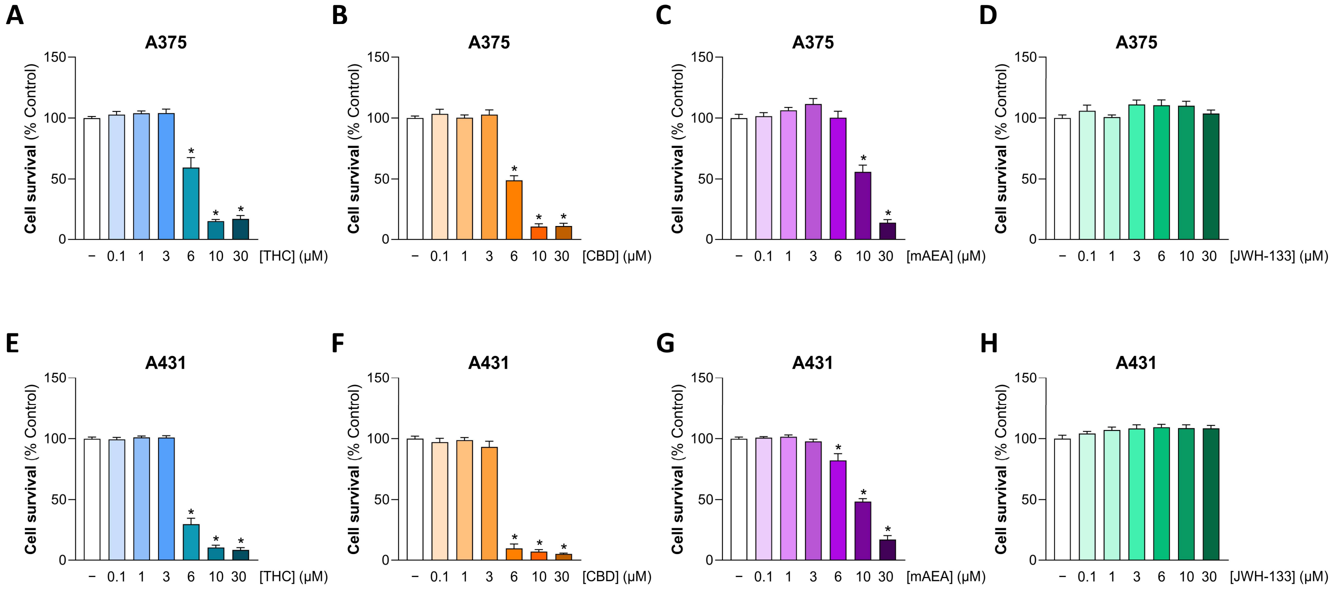

Supplementary Figure S1. Concentration-dependent effects of THC (**A**,**E**), CBD (**B**,**F**), mAEA (**C**,**G**), and JWH-133 (**D**,**H**) on cell number of A375 and A431 cells. Cells were incubated with the respective cannabinoid at the indicated concentrations for 48 h. The values given are based on crystal violet assays. All percentage values shown refer to the respective vehicle control, which was set to 100%. The data are mean values ± SEM of n = 9 per group from 3 independent experiments (**B**,**E**,**F**) or n = 11–12 from 4 independent experiments (**A**,**C**,**D**,**G**,**H**). * $p \leq 0.05$ vs. corresponding vehicle control; one-way ANOVA with Dunnett's post hoc test.

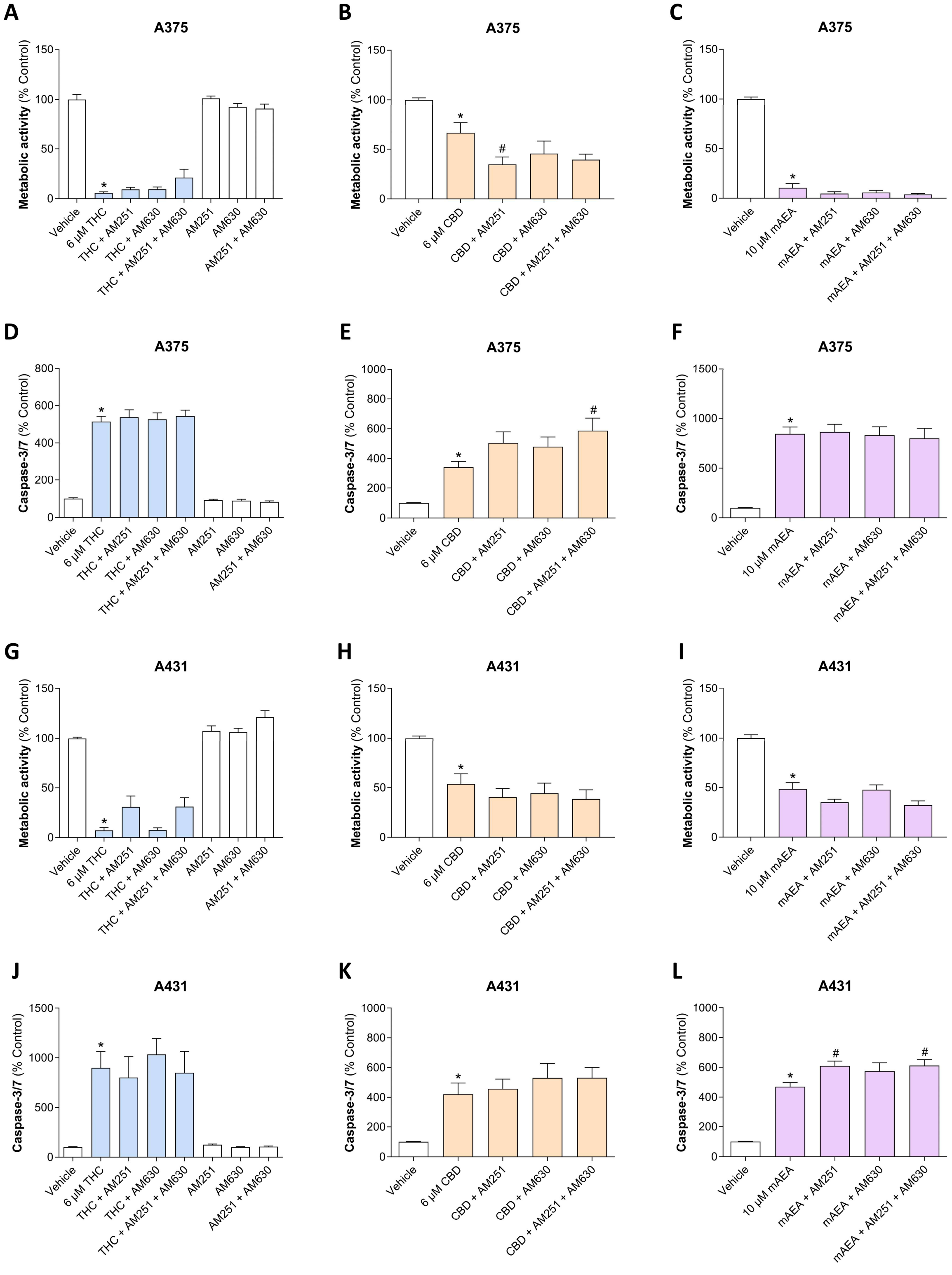

Supplementary Figure S2. Influence of the CB_1 antagonist AM251 and the CB_2 antagonist AM630 on the decrease in metabolic activity as well as on the increase in caspase-3/7 activity mediated by THC (6 µM), CBD (6 µM), or mAEA (10 µM) in A375 (**A**–**F**) and A431 cells (**G**–**L**). Cells were pre-treated with AM251 (1 µM), AM630 (1 µM), a combination of both antagonists (each at 1 µM) or vehicle for 1 h, followed by a 48-h (A375 cells) or 24-h (A431 cells) co-incubation with the indicated concentrations of THC, CBD or mAEA or its vehicle. All percentage values shown refer to the respective vehicle control, which was set to 100%. The data are mean values ± SEM of $n = 9$ from 3 independent experiments. * $p \leq 0.05$ vs. corresponding vehicle control; # $p \leq 0.05$ vs. corresponding CBD- or mAEA-treated group; one-way ANOVA with Bonferroni's post hoc test.

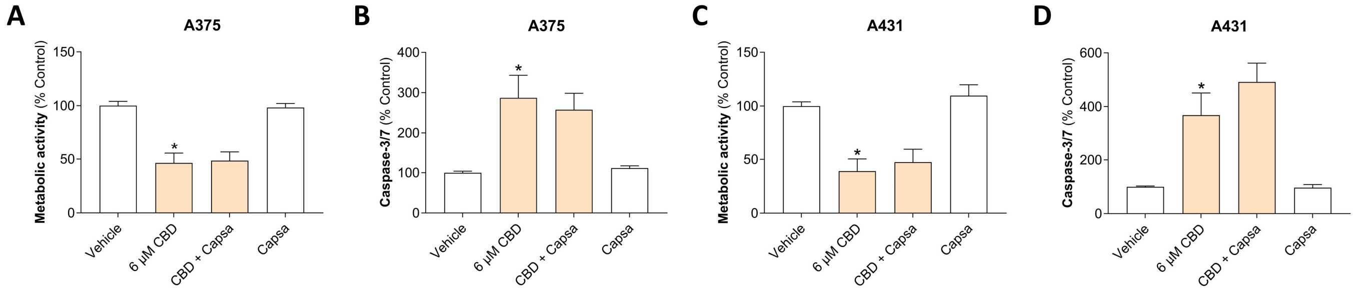

Supplementary Figure S3. Influence of the TRPV1 antagonist capsazepine (Capsa) on the decrease in metabolic activity and the increase in caspase-3/7 activity mediated by CBD (6 µM) in A375 (**A**,**B**) and A431 cells (**C**,**D**). Cells were pre-treated with capsazepine (1 µM) or vehicle for 1 h, followed by a 48-h (A375 cells) or 24-h (A431 cells) co-incubation with CBD or its vehicle. All percentage values shown refer to the respective vehicle control, which was set to 100%. The data are mean values ± SEM of n = 9 from 3 independent experiments. * $p \leq 0.05$ vs. corresponding vehicle control; one-way ANOVA with Bonferroni's post hoc test.

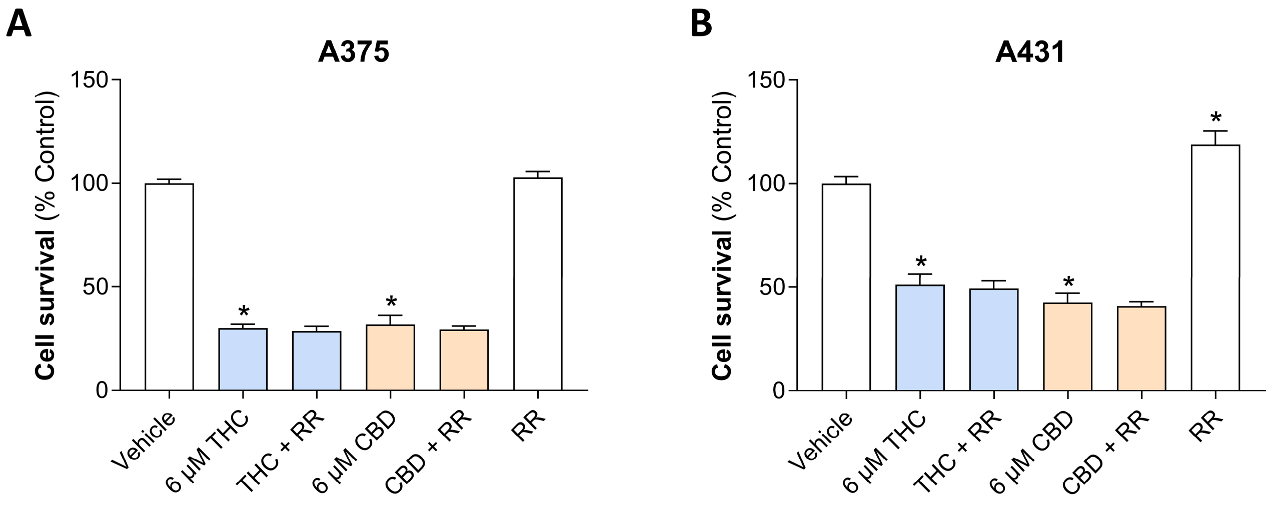

Supplementary Figure S4: Influence of the broad-spectrum TRP channel inhibitor ruthenium red (RR) on the decrease in cell number mediated by THC or CBD in A375 (**A**) and A431 cells (**B**). Cells were pre-treated with ruthenium red (10 µM) or vehicle for 1 h, followed by a 48-h (A375 cells) or 24-h (A431 cells) co-incubation with 6 µM THC or 6 µM CBD or its vehicle. All percentage values shown refer to the respective vehicle control, which was set to 100%. The data are mean values ± SEM of n = 9 from 3 independent experiments. * $p \leq 0.05$ vs. corresponding vehicle control; one-way ANOVA with Bonferroni's post hoc test.

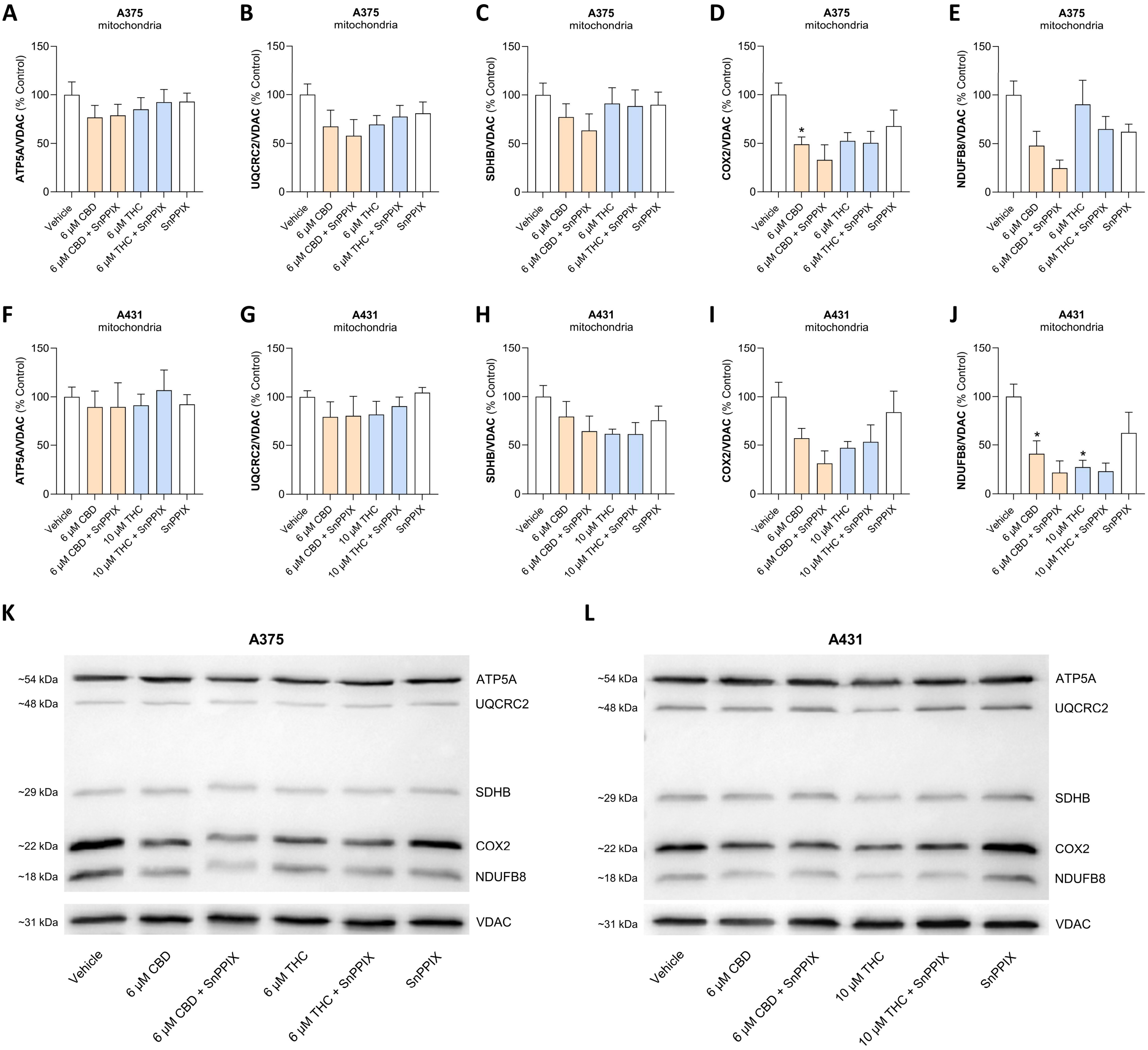

Supplementary Figure S5. Investigation of the impact of SnPPIX on THC and CBD effects regarding the concentrations of subunits of mitochondrial respiratory chain complexes of A375 (**A**–**E**,**K**) and A431 cells (**F**–**J**,**L**). Cells were pretreated with SnPPIX (25 µM) or its vehicle for 1 h, followed by a 24-h co-incubation with the indicated concentrations of THC or CBD or its vehicle. Thereafter, the corresponding proteins in the mitochondrial fractions were determined using Western blot. The values given in the bar charts are based on the densitometric analyses of blots. Mitochondrial proteins were normalized to VDAC. All percentage values shown refer to the respective vehicle control, which was set to 100%. The blots shown (**K**,**L**) are representative. The data are mean values ± SEM of n = 5 independent experiments. * $p \leq 0.05$ vs. corresponding vehicle control; one-way ANOVA with Bonferroni's post hoc test.
